# A Systematic Review of Natural Language Processing Techniques for Early Detection of Cognitive Impairment

**DOI:** 10.1016/j.mcpdig.2025.100205

**Published:** 2025-03-05

**Authors:** Ravi Shankar, Anjali Bundele, Amartya Mukhopadhyay

**Affiliations:** a Medical Affairs–Research Innovation and Enterprise, Alexandra Hospital, National University Health System, Singapore; b Division of Respiratory and Critical Care Medicine, Department of Medicine, National University Health System, Singapore

## Abstract

**Objective:**

To systematically evaluate the effectiveness and methodologic approaches of natural language processing (NLP) techniques for early detection of cognitive decline through speech and language analysis.

**Methods:**

We conducted a comprehensive search of 8 databases from inception through August 31, 2024, following Preferred Reporting Items for Systematic Reviews and Meta-Analyses guidelines. Studies were included if they used NLP techniques to analyze speech or language data for detecting cognitive impairment and reported diagnostic accuracy metrics. Two independent reviewers (R.S. and A.B.) screened articles and extracted data on study characteristics, NLP methods, and outcomes.

**Results:**

Of 23,562 records identified, 51 studies met inclusion criteria, involving 17,340 participants (mean age, 72.4 years). Combined linguistic and acoustic approaches achieved the highest diagnostic accuracy (average 87%; area under the curve [AUC], 0.89) compared with linguistic-only (83%; AUC, 0.85) or acoustic-only approaches (80%; AUC, 0.82). Lexical diversity, syntactic complexity, and semantic coherence were consistently strong predictors across cognitive conditions. Picture description tasks were most common (n=21), followed by spontaneous speech (n=15) and story recall (n=8). Crosslinguistic applicability was found across 8 languages, although language-specific adaptations were necessary. Longitudinal studies (n=9) reported potential for early detection but were limited by smaller sample sizes (average n=159) compared with cross-sectional studies (n=42; average n=274).

**Conclusion:**

Natural language processing techniques show promising diagnostic accuracy for detecting cognitive impairment across multiple languages and clinical contexts. Although combined linguistic-acoustic approaches appear most effective, methodologic heterogeneity and small sample sizes in existing studies suggest the need for larger, standardized investigations to establish clinical utility.

Cognitive decline, encompassing conditions, such as mild cognitive impairment (MCI), Alzheimer disease (AD), and other forms of dementia, represents a major global health challenge across a wide spectrum of medical conditions.^1^^,^^2^ As the world’s population ages, the prevalence of these conditions is expected to rise dramatically.^3^ However, cognitive decline is not limited to age-related conditions and can occur in various other medical contexts, such as cancer,^4^ stroke,^5^ and psychiatric disorders.^6^ Early detection of cognitive decline is crucial for timely intervention, disease management, and care planning.^7^ However, current diagnostic methods often rely on extensive neuropsychological testing and neuroimaging, which can be time-consuming, costly, and impractical for large-scale screening.^8^, ^9^, ^10^, ^11^

Recent advancements in natural language processing (NLP) techniques have opened new avenues for detecting subtle linguistic changes that may serve as early markers of cognitive impairment.^12^ Natural language processing methods can analyze various aspects of speech and language, such as lexical diversity, syntactic complexity, semantic coherence, and acoustic features, to identify patterns indicative of cognitive decline.^13^, ^14^, ^15^, ^16^, ^17^, ^18^, ^19^, ^20^ The potential of NLP-based approaches lies in their ability to provide objective, automated, and noninvasive assessment of cognitive function from readily available speech and language data.

This systematic review aimed to synthesize the current state of research on NLP techniques for early detection of cognitive decline. We seek to provide a comprehensive overview of the study designs, participant characteristics, speech elicitation methods, and NLP techniques used in the field. By comparing the key findings, accuracy metrics, and limitations of existing studies, we aimed to identify the most promising approaches and highlight areas for future research. Additionally, we explored the potential for early detection, monitoring, and clinical applicability of NLP methods in the context of cognitive impairment across various health conditions.

## Methods

This systematic review was conducted according to the Preferred Reporting Items for Systematic Reviews and Meta-Analyses guidelines.^21^ The review protocol was registered with the International Prospective Register of Systematic Reviews under the registration number CRD42024592875.

### Search Strategy

We conducted a comprehensive search of PubMed, Web of Science, Embase, CINAHL, MEDLINE, The Cochrane Library, PsycINFO, and Scopus databases from inception to August 2024. The search strategy was adapted for each database. The following search string was used as a basis:

*((“natural language processing” OR “computational linguistics” OR “text mining” OR “speech analysis” OR “language analysis” OR “discourse analysis” OR “linguistic feature∗”) AND (“cognitive impairment” OR “cognitive decline” OR “dementia” OR “Alzheimer∗” OR “mild cognitive impairment” OR “MCI” OR “neurodegenerative” OR “neurological disorder∗”) AND (“detection” OR “diagnosis” OR “classification” OR “prediction” OR “screening” OR “early identification”) AND (“speech” OR “language” OR “spontaneous speech” OR “discourse” OR “narrative”))*

The search was limited to English-language articles. Additional relevant studies were identified through manual screening of reference lists and citation searching.

### Eligibility Criteria

Studies were included if they met the following criteria:

1. Used NLP techniques to analyze speech or language data for the purpose of detecting cognitive impairment
2. Included participants with MCI, AD, dementia, or other forms of neurocognitive health conditions
3. Reported accuracy metrics or diagnostic performance of the NLP-based approach
4. Published in a peer-reviewed journal or conference proceedings

Studies were excluded if they:

1. Focused solely on acoustic or paralinguistic features without linguistic analysis
2. Used only structured language tasks (eg, verbal fluency tests) without analyzing spontaneous speech or discourse
3. Did not include a cognitive impairment group or did not report diagnostic accuracy
4. Were case studies, reviews, or commentaries without original data
5. Had the wrong study setting, outcomes, article type, study design, or population
  • Study setting: Study settings that did not involve direct speech/language assessment (eg, medical record reviews only, online surveys without speech components)
  • Outcomes: Outcomes not related to cognitive function assessment (eg, studies focused only on speech technology development without clinical applications)
  • Article type: Editorials, letters, conference abstracts without full text, or study protocols without results
  • Study design: Single case reports or studies without appropriate control/comparison groups, also study designs that were purely theoretical or methodological without empirical data
  • Population: Study populations that did not include participants with or at risk for cognitive impairment
6. Were not available as full text in English
7. Did not investigate cognitive decline using NLP from speech data (studies using electronic health records or other text data were excluded)

### Study Selection and Data Extraction

The study selection process is summarized in the Preferred Reporting Items for Systematic Reviews and Meta-Analyses flow diagram (Figure). The initial search yielded 23,562 records from databases and 811 additional records from other sources. After removing 19,340 duplicates, 5033 records were screened by title and abstract. Of these, 104 articles were sought for retrieval, and all 104 were assessed for eligibility. A total of 53 studies were excluded based on the predefined exclusion criteria, leaving 51 studies to be included in the review.

**Figure:**
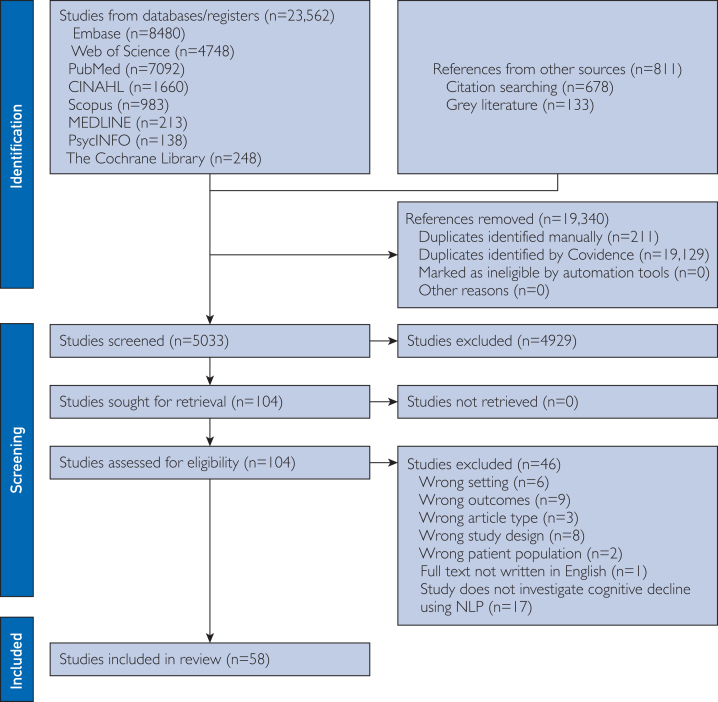
Preferred Reporting Items for Systematic Reviews and Meta-Analyses flow diagram.

Two reviewers (R.S. and A.B.) independently screened the titles and abstracts of retrieved articles, followed by full-text review of potentially eligible studies. Disagreements were resolved through discussion or consultation with a third reviewer. Data were extracted using a standardized form, including information on study design, participant characteristics, speech elicitation methods, NLP techniques, key findings, accuracy metrics, and limitations (Supplemental Table 1, available online at https://www.mcpdigitalhealth.org/). The data extraction process was designed to capture key information about each study, enabling a comprehensive analysis and synthesis of the current state of research on NLP techniques for early detection of cognitive decline. The extracted data were used to generate summary tables and comparative analyses, which are presented in the Results section of this systematic review.

### Quality Assessment

The quality of included studies was assessed using a customized quality assessment tool adapted from the Newcastle-Ottawa Scale and the QUADAS-2 tool. The quality assessment evaluated 3 key domains with defined parameters. The participant selection domain examined clear inclusion/exclusion criteria, representative sampling methods, adequate sample size justification, appropriate matching of control groups, and demographic balance between groups. The speech data collection domain assessed standardized data collection protocols, appropriate recording conditions and equipment, consistent elicitation methods, adequate quality control measures, and complete documentation of collection procedures. The analysis methods domain evaluated appropriate statistical/NLP techniques, validated measurement tools, adequate handling of missing data, proper crossvalidation procedures, and transparent reporting of results. Each domain’s parameters were used to determine risk levels, with studies meeting all or most parameters rated as low risk, those with some limitations as moderate risk, and those with significant methodologic concerns as high risk. Each study was evaluated on 3 domains: participant selection, speech data collection, and analysis methods. Within each domain, studies were rated as having low, moderate, or high risk of bias. An overall study quality rating was then assigned based on the combination of domain-level ratings, ranging from low to high quality.

### Data Synthesis and Analysis

Extracted data were synthesized using narrative and tabular formats. Descriptive statistics were used to summarize study characteristics, participant demographic characteristics, and accuracy metrics. Comparative analyses were conducted to identify patterns and trends across studies, as well as to highlight methodological differences and research gaps. Owing to the heterogeneity of study designs, NLP techniques, and outcome measures, a meta-analysis was not feasible.

## Results

### Study Characteristics

The 51 included studies were published between 2011 and 2024, with a notable increase in publications in recent years. All studies were published in English, although several analyzed speech data in other languages including Chinese, Japanese, Spanish, Italian, Greek, Turkish, French, and Cantonese. The study designs were predominantly observational, with a mix of cross-sectional (n=42) and longitudinal (n=9) approaches. Sample sizes ranged from 4 to 25,192 participants, with an average of 340 participants per study.

Table 1^4^, ^5^, ^6^^,^^14^^,^^16^^,^^17^^,^^19^^,^^20^^,^^22^, ^23^, ^24^, ^25^, ^26^, ^27^, ^28^, ^29^, ^30^, ^31^, ^32^, ^33^, ^34^, ^35^, ^36^, ^37^, ^38^, ^39^, ^40^, ^41^, ^42^, ^43^, ^44^, ^45^, ^46^, ^47^, ^48^, ^49^, ^50^, ^51^, ^52^, ^53^, ^54^, ^55^, ^56^, ^57^, ^58^, ^59^, ^60^, ^61^, ^62^, ^63^, ^64^ provides an overview of the key characteristics of included studies, including country, language of speech data, study design, sample size, participant groups, speech elicitation method, NLP methods, main findings, and limitations. Speech elicitation methods varied, with picture description tasks, particularly the Cookie Theft picture from the Boston Diagnostic Aphasia Examination, being the most frequent (n=21). Other methods included story recall (n=8), spontaneous speech (n=15), neuropsychological test interviews (n=6), and conversations with virtual agents or chatbots (n=8). The studies usedemployed a wide range of NLP techniques including acoustic and linguistic feature extraction.

**Table 1:** Overview of Studies on Speech and Language Analysis in Cognitive Impairment

Country		Language	Design	Sample size	Participant groups	Speech elicitation method	NLP methods	Reference standard and Test scores	Main findings and test correlations	Limitations
Huang et al,^14^ 2024	China	Chinese	ML and computational modeling	92	AD, MCI, HC	Cookie theft picture description	ASR, NLP analysis	MoCA-B: AD: 10.03 (3.200), MCI: 19.98 (2.616), HC: 24.73 (2.251)	Accuracy 80.77% (SVM) and 80.43% (RF) in HC vs AD classification; linguistic features reported significant correlation with MoCA-B scores, particularly lexical diversity and acoustic features	Relatively small data set, limited to Chinese language
Diaz-Asper et al,^22^ 2022	USA	English	Cross-sectional	91	AD, aMCI, HC	Semantic word fluency, free speech	Manual transcription, NLP analysis	MMSE: AD: 23.75 (0.51), aMCI: 28.22 (0.30), HC: 29.56 (0.12)	AUC 0.90 for HC vs AD classification; classification performance strongly correlated with MMSE scores across diagnostic groups	Small sample size, highly educated sample
Amini et al,^23^ 2023	USA	English	Longitudinal cohort	1084	Normal, MCI, dementia	Neuropsychologic test interviews	ASR, universal sentence encoder	Neuropsychologic test battery, scores not specified	AUC 0.926 for dementia detection; high correlation between NLP features and neuropsychologic test battery scores	Limited MCI samples for manual analysis
Beltrami et al,^16^ 2018	Italy	English	Experimental	96	AD, aMCI, mdMCI, HC	Picture description, working day, dream recall	Manual transcription, NLP analysis	MMSE ≥18 for inclusion, specific scores not reported	Acoustic features most sensitive to cognitive decline and reported strongest correlation with MMSE scores; specific correlation coefficients not reported	Small sample size, education level differences
Fristed et al,^24^ 2022	UK	English	Prospective cohort	133	CU, MCI/mild AD	Story recall task	ASR, NLP libraries, ParaBLEU model	Story recall test scores, not specified by group	AUC 0.85 for MCI/mild AD detection; performance correlated with story recall test scores	Limited to British English speakers, potential practice effects
Mahajan and Baths,^25^ 2021	India	English	Case-control	164	AD, MCI, HC	Cookie Theft picture description	n-Gram language models, SVD	MMSE, scores not specified	AUC 0.83 for AD vs HC using 5-gram model; correlation with MMSE scores through linguistic features	Limited data set size, focus on single task
Nasreen et al,^26^ 2021	Greece	Greek	Case-control	30	MCI, HC	Spontaneous written speech	POS tagging, dependency parsing	Clinical diagnosis only	AUC 0.78 for MCI detection using keystroke features; correlations based on clinical diagnosis	Small sample size, specific educational levels required
Haulcy and Glass,^27^ 2021	USA	English	ML and computational modeling	156	AD, HC	Cookie Theft picture description	BERT embeddings, acoustic features	Clinical diagnosis only	85.4% accuracy using SVM on BERT embeddings; validated against clinical diagnosis	Small test set, dependency on transcripts
Igarashi and Nihei,^28^ 2022	Japan	Japanese	Cross-sectional	60	Healthy older adults, MCI	Episodic task, picture description, animation description	BERT pretrained Japanese model	Clinical diagnosis only	F1 score 0.891 with data augmentation; correlated with clinical diagnostic criteria	Small sample size, especially for MCI group
Kleiman and Galvin,^29^ 2024	USA	English	Cross-sectional	53	MCI, HC	Narrative recall, picture description, free response	ASR, NLP feature extraction	Clinical diagnosis only	AUC 0.791 for postpause features model; correlated with clinical diagnosis	Limited sample size, age differences between groups
Robin et al,^30^ 2023	USA	English	Longitudinal	130	Prodromal to mild AD	Clinical dementia rating interview	ASR, NLP for feature extraction	Clinical dementia rating	Composite score reported similar effect sizes as clinical end points; validated against CDR scores	Homogeneous sample, lack of healthy control group
Horigome et al,^31^ 2022	Japan	Japanese	Prospective observational	432	Dementia, non-dementia	Unstructured free conversation	Custom vector representation	MMSE—dementia: 16.4±4.8, non-dementia: 28.6±1.8 CDR—dementia: 1.3±0.7, non-dementia: 0.1±0.2	AUC 0.935 for dementia classification; strong correlations with both MMSE and CDR scores	Relatively small sample size, potential confounding factors
Liu et al,^32^ 2021	China	English	ML and computational modeling	498	AD, controls	Cookie Theft picture description	Transformer encoder, feature purification network	Clinical diagnosis only	93.5% accuracy on Pitt data set; validated against clinical diagnosis	Limited reporting of limitations and participant characteristics
Ntracha et al,^33^ 2020	Greece	Greek	Case-control	23	MCI, HC	Semistructured interview	POS tagging, dependency parsing	Clinical diagnosis only	AUC 0.78 for combined features; correlated with clinical diagnostic criteria	Small cohort size, specific educational levels required
Badal et al,^34^ 2024	USA	English	Longitudinal	71	Older adults without known demented	Semistructured qualitative interview	ASR, NLP feature extraction	Clinical diagnosis only	F1 scores 0.73-0.86 for combined features; validated against clinical diagnosis	Small sample size, homogeneous sample
Hernández-Domínguez et al,^35^ 2018	Canada, Mexico	English	Cross-sectional	517	AD, MCI, HC	Cookie Theft picture description	Manual transcription, NLP analysis	MMSE, scores not reported	AUC 0.79 for AD vs HC; correlation with MMSE through linguistic features	Small MCI sample, lack of acoustic analysis
Orimaye et al,^36^ 2018	USA	English	ML and computational modeling	236	AD, MCI, HC	Cookie Theft picture description	n-Gram language models, SVD	Clinical diagnosis only	AUC 0.83 for AD vs HC using deep language space neural network; validated against clinical diagnosis	Limited demographic information provided
Parsapoor et al,^37^ 2023	Canada	English	ML and computational modeling	22	Dementia, HC	Picture description, story recall	ASR, NLP feature extraction	Clinical diagnosis only	Up to 93% F1 score for ML models; correlation with clinical diagnosis	Small sample size, imbalanced data sets
Wang et al,^38^ 2021	China	Chinese	Case-control	110	MCI, HC	Picture description, semantic fluency, sentence repetition	ASR, NLP feature extraction	Clinical diagnosis only	94%-96% accuracy for combined tasks; model performance validated against clinical diagnostic groups	Relatively small sample size, limited to Mandarin speakers
Runde et al,^19^ 2024	USA	English	ML and computational modeling	521	AD, MCI, control	Cookie Theft picture description	GPT embeddings, ASR	Clinical diagnosis only	Up to 0.99 accuracy for AD vs control using GPT embeddings; validated against clinical diagnosis	Limited samples for MCI and possible AD groups
Lindsay et al,^39^ 2021	USA	English	ML	154	AD, HC	Cookie Theft picture description	Manual transcription, NLP analysis	Clinical diagnosis only	Classification accuracy: English baseline 69.7%, with generalizable features 76.4%; validated against clinical diagnosis	Age and education not matched between groups
Mirheidari et al,^5^ 2024	UK	English	Experimental	55	Stroke survivors	Conversation with IVA	ASR, NLP analysis	Clinical diagnosis only	Sensitivity 0.75, specificity 0.73 for cognitive impairment detection in stroke survivors; correlations with clinical assessments	Small sample size, predominantly mild stroke cases
de Arriba-Pérez et al,^40^ 2023	Spain	Spanish	Experimental	30	Elderly with absent (43%), mild (40%), and severe (17%) cognitive impairment	Conversation with entertainment chatbot	ASR via Google Voice SDK, NEC, MCR database analysis, GainRatioAttributeEval feature selection	Clinical diagnosis only	Decision Tree accuracy 86.67%, F measure 88.20%, recall 83.30%; validated against clinical diagnosis	Small sample size, Spanish language only, stress/focus as confounders
Li et al,^41^ 2024	USA	English	ML and computational modeling	343	AD, HC	Cookie Theft picture description	ASR models, BERT classification	Clinical diagnosis only	ASR errors improved dementia classification with AUC of 0.903; correlation with clinical diagnosis	Small data set, limited to American English speakers
Skirrow et al,^42^ 2022	UK, USA	English	Longitudinal case-control	151	CU, MCI/mild AD	Story recall	ASR, NLP analysis	Story recall task scores	AUC 0.86 for detecting MCI/mild AD; correlation with story recall performance	Small sample size, lack of racial diversity
de Arriba-Pérez et al,^43^ 2024	Spain	English	Experimental	42	Cognitive impairment present/absent	Free dialogs with AI assistant	GPT 3.5-turbo for feature extraction	Clinical diagnosis only	98.47% accuracy using random forest; validated against clinical diagnosis of cognitive impairment	Small sample size, potential biases of language models
Roark et al,^44^ 2011	USA	English	Case-control	74	MCI, HC	Story recall	Manual transcription, NLP analysis	Clinical diagnosis only	AUC 0.861 for MCI detection using spoken language–derived measures; validated against clinical diagnosis	Small sample size, potential overfitting
Amini et al,^45^ 2024	USA	English	Longitudinal prospective cohort	166	Stable MCI, progressive MCI	Neuropsychologic test interview	ASR, NLP analysis	Clinical diagnosis only	78.2% accuracy in participant’s progression to AD; correlation with progression to clinical AD diagnosis	Small sample size, limited to English language
de Arriba-Pérez and García-Méndez,^46^ 2024	Spain	English	Experimental	44	Mental deterioration present/absent	Free dialog with chatbot	GPT 3.5-turbo for feature extraction	Clinical diagnosis only	77.70% accuracy using ARFC model for mental deterioration detection; validated against clinical diagnosis	Limited information on participant selection
Šubert et al,^47^ 2023	Czech Republic	Czech	Case-control	240	MS, HC	Spontaneous discourse	ASR, NLP analysis	Clinical diagnosis only	AUC 0.70 for MS detection using lexical and syntactic features; correlation with MS diagnosis	High word error rate in automated transcription
Fraser et al,^48^ 2016	Canada	English	ML and computational modeling	264	AD, HC	Cookie Theft picture description	Manual transcription, NLP analysis	Clinical diagnosis only	81.92% accuracy for AD detection using linguistic features; validated against clinical diagnosis	Age and education not matched between groups
Kim et al,^49^ 2024	USA	English	ML and computational modeling	169	aMCI, naMCI	Written picture description	NLP analysis, BERT embeddings	Clinical diagnosis only	90% accuracy for aMCI vs naMCI classification using BERT embeddings; correlation with clinical diagnosis	Small data set, especially for naMCI group
Gómez-Valadés et al,^50^ 2024	Spain	Spanish	ML and computational modeling	141	HC, heterogeneous MCI, Stable MCI	Semantic fluency tests: animals, clothes, plants, vehicles for 60 s each	NLP for automatic scoring, feature selection using GainRatioAttributeEval algorithm, 6 ML models (eg, random forest and SVM)	Clinical tests, scores not reported	Best combination achieved 86.67% accuracy with random forest (F1 scores: RF=0.694); correlation with clinical tests	Small sample size, monolingual Spanish cohort, mild symptom overlap between groups
Hajjar et al,^51^ 2023	USA	English	Observational	206	CU, MCI	Multiple speech tasks	ML, NLP analysis	Clinical diagnosis only	AUC 0.80 for MCI detection using lexical-semantic features; validated against clinical diagnosis	Relatively small sample size, potential misclassification issues
Sangchocanonta et al,^52^ 2021	Thailand	Thai	Cross-sectional	90	AD, MCI, HC	Picture description	POS tagging, ML classification	Clinical diagnosis only	AUC 0.8480 for best model using POS tagging; correlation with clinical diagnosis	Small sample size, age differences between groups
Yan et al,^53^ 2024	USA	English	ML and computational modeling	34	Older adults	Natural interactions with Amazon Alexa	Rule–based NLP model	MoCA mean 23.47 (SD 3.65)	85.49% agreement between manual and MR-NLP coding; correlation with MoCA scores	Small sample size, limited diversity
Kalpana Chowdary et al,^54^ 2025	India	English	Prospective observational	9	Elderly populations	Conversational interactions with robot	ASR, NLP analysis	Clinical diagnosis only	Differences in interaction patterns between cognitive groups; validated against clinical assessment	Very small sample size, limited details on analysis methods
Liang et al,^55^ 2022	USA	English	Experimental	40	MCI, HC	Voice commands to digital assistant	ASR, NLP analysis	Clinical diagnosis only	68% accuracy using early fusion of all features in voice-assistant commands; correlation with clinical diagnosis	Small sample size, limited demographic representation
Soroski et al,^56^ 2022	Canada	English	Cross-sectional	149	AD, MCI, SMC, HC	Picture description, reading, recall	ASR, NLP analysis	Clinical diagnosis only	AUC ranging from 0.503 to 0.755 for various tasks; validated against clinical diagnosis	Small sample size, potential interrater variability
Khodabakhsh et al,^57^ 2015	Turkey	Turkish	Case-control	79	AD, HC	Unstructured conversational interview	Manual transcription, NLP analysis	Clinical diagnosis only	83.5% accuracy using silence ratio with SVM; correlation with clinical diagnosis	Limited number of patients, especially patients with AD
Sigona et al,^58^ 2025	Italy	Italian	Cross-sectional	216	People with dementia	Naturalistic conversations	Morphosyntactic analysis, feature selection	MMSE ranges: severe: 0-9, moderate: 10-20, mild: 21-26	Hapax count, noun/verb ratio, subjunctive mood usage were key features; strong correlation with MMSE ranges	Limited number of participants in mild category, lack of healthy controls
Anmella et al,^6^ 2024	Spain, USA	Catalan, Spanish, English	Observational	76	Bipolar disorder (manic, depressive, euthymic)	Multiple speech tasks	ASR, NLP feature extraction	Clinical diagnosis only	Preliminary clinical and demographic data reported correlation patterns with bipolar disorder phases	Small sample size, potential misclassification issues
Yeung et al,^20^ 2021	Canada	English	Cross-sectional	30	AD, MCI, HC	Cookie Theft picture description	Manual transcription, NLP analysis	MMSE: AD: 18 (1.60), MCI: 24 (1.95), HC: 29 (0.89)	Word-finding difficulty and incoherence most useful in distinguishing groups; strong correlation with MMSE scores	Small sample size, potential systematic biases
Reeves et al,^59^ 2020	USA	English	Prospective observational cohort study	56	Normal cognition, impaired not MCI cognitively impaired (not MCI), MCI, dementia	Video scene description	Manual transcription, NLP analysis	Clinical diagnosis only	Narrative description scores declined with increasing cognitive impairment; validated against clinical diagnosis	Small sample size, higher education levels than national average
Stille et al,^60^ 2019	Germany	English	Computational modeling	NA	NA	Simulated picture naming, word repetition	Neural engineering framework	Not applicable (computational model)	Effects of neural ablation on word production performance; theoretical model without clinical correlation	Simplified input/output processes, limited vocabulary size
Kong et al,^61^ 2023	Hong Kong	Cantonese	Cross-sectional	104	People with dementia	Personal narrative, picture description	Manual coding and analysis	Clinical diagnosis only	Global coherence predicted episodic autobiographical memory; correlation with clinical assessment	Small sample size, limited to Cantonese-speaking PWD
Pistono et al,^62^ 2016	France	English	Case-control	30	MCI due to AD, HC	Autobiographical discourse	Manual transcription, pause analysis	Clinical diagnosis only	Patients produced more between-utterance pauses than controls; validated against clinical diagnosis	Small sample size, no limitations reported
Ferrario et al,^63^ 2022	Switzerland, USA	English	Cross-sectional	98	Healthy older adults	Naturalistic observation	Manual transcription, NLP analysis	Clinical diagnosis only	Improvement in MSE for cognitive task prediction; correlation with cognitive performance	Limited sample size, no detection of changes over time
Panesar and Pérez Cabello de Alba,^17^ 2023	UK, Spain	English	Experimental	4	Varying cognitive decline	Multiple speech tasks	Manual feature extraction	GDS stages 3-6, scores not reported	Model results correlated with clinical GDS ratings; direct correlation with GDS stages	Very small sample size, manual analysis
Williams et al,^64^ 2021	USA	English	Feasibility study	13	Men receiving ADT for prostate cancer	Clinical interview, prompt question	Manual transcription and coding	Clinical diagnosis only	Correlations between psycholinguistic and neurocognitive measures in cancer survivors	Small sample size, limited to specific patient group
Aramaki et al,^4^ 2019	Japan	Japanese	Prospective cohort	116	Cancer patients	Interview	ASR, NLP analysis	HDS-R, scores not reported	Type-token ratio reported highest correlation with HDS-R scores in cancer patients	Small sample size, limited to Japanese-speaking cancer patients

AD, Alzheimer disease; ADT, androgen deprivation therapy; aMCI, amnestic mild cognitive impairment; ARFC, adaptive random forest classifier; ASR, automatic speech recognition; AUC, area under the curve; BERT, bidirectional encoder representations from transformers; CDR, clinical dementia rating; CI, cognitive impairment; CU, cognitively unimpaired; GDS, global deterioration scale; GPT, generative pretrained transformer; HC, healthy control; HDS-R, Hasegawa’s Dementia Scale-Revised; IVA, intelligent virtual agent; MCI, mild cognitive impairment; mdMCI, multiple-domain mild cognitive impairment; ML, machine learning; MMSE, mini-mental state examination; MoCA, Montreal cognitive assessment; MR-NLP, modified rule–based natural language processing; MS, multiple sclerosis; MSE, mean squared error; naMCI, nonamnestic mild cognitive impairment; NLP, natural language processing; POS, part-of-speech; PWD, people with dementia; SMC, subjective memory complaint; SVD, singular value decomposition; SVM, support vector machine.

The main findings found the potential of NLP methods in detecting cognitive impairment, with reported accuracies and area under the curve (AUC) values ranging from 0.503 to 0.99. However, many studies had limitations, such as small sample sizes, lack of diversity in participant groups, and potential biases related to language, education, or cultural factors.

### Participant Characteristics

The included studies involved 17,340 participants, with an average age of 72.4 years (range, 44-98 years). Most participants were cognitively healthy controls (n=7525), followed by individuals with AD (n=4730), MCI (n=4526), and other forms of cognitive impairment (n=559). Participants had a wide range of comorbid health conditions, including Parkinson disease (PD), multiple sclerosis, stroke, cancer, bipolar disorder, and liver disease. Most studies matched participant groups by age and education level, although some differences were noted.

### Speech Elicitation Methods

A variety of speech elicitation methods were used across studies, ranging from highly structured tasks to spontaneous discourse. Picture description tasks, particularly the Cookie Theft picture from the Boston Diagnostic Aphasia Examination, were the most common (n=21). Other methods included story recall (n=8), spontaneous speech (n=15), neuropsychological test interviews (n=6), and conversations with virtual agents or chatbots (n=8).

Table 2^5^^,^^6^^,^^14^^,^^16^^,^^19^, ^20^, ^21^, ^22^, ^23^, ^24^, ^25^, ^26^, ^27^, ^28^, ^29^^,^^31^^,^^32^^,^^34^^,^^35^^,^^37^^,^^40^, ^41^, ^42^^,^^44^, ^45^, ^46^, ^47^, ^48^^,^^51^, ^52^, ^53^^,^^55^, ^56^, ^57^, ^58^, ^59^ compares the advantages, disadvantages, and potential impact of different speech elicitation tasks used in the included studies. Picture description tasks were found to be the most standardized and easy to administer, assessing multiple cognitive domains. However, they are limited to visual processing and descriptive abilities. Spontaneous speech tasks, although more natural and reflective of real-world communication, are difficult to standardize and yield variable content. Conversations with virtual agents or chatbots show promise for remote, continuous monitoring of cognitive health but require further validation.

**Table 2:** Comparison of Speech Elicitation Tasks in Cognitive Assessment

Task description	Advantages	Disadvantages	Potential impact	Studies
Picture description	Standardized, easy to administer, assesses multiple cognitive domains	Limited to visual processing and descriptive abilities	Early detection of cognitive decline, language impairment assessment	^16^^,^^19^^,^^20^^,^^24^^,^^25^^,^^27^^,^^28^^,^^32^^,^^35^^,^^37^^,^^41^^,^^48^^,^^52^^,^^59^
Story recall	Assesses memory and language abilities, can be standardized	May be influenced by education and cultural background	Memory impairment detection, language assessment	^24^^,^^42^^,^^44^
Spontaneous speech	Natural, reflects real-world communication	Difficult to standardize, variable content	Detecting subtle changes in language use, pragmatic deficits	^5^^,^^14^^,^^26^^,^^31^^,^^34^^,^^47^^,^^56^, ^57^, ^58^
Neuropsychological test interviews	Comprehensive, assesses multiple cognitive domains	Time-consuming, requires trained administrators	Detailed cognitive profiling, early detection of various impairments	^23^^,^^45^^,^^51^
Voice commands	Easy to administer, reflects real-world technology use	Limited scope of language use, potential technological barriers	Monitoring cognitive health through everyday technology	^53^^,^^55^
Conversational artificial intelligence interactions	Natural interaction, can be done remotely	Requires technology access, potential for misunderstandings	Continuous monitoring of cognitive health	^6^^,^^29^^,^^40^^,^^46^
Video scene description	Dynamic stimuli, assesses visual processing and narrative skills	Requires equipment, may be influenced by visual acuity	Detecting subtle changes in cognitive processing and language	^59^

### NLP Techniques

The NLP techniques used in the included studies can be broadly categorized into linguistic feature extraction, acoustic feature analysis, and combined approaches. Linguistic features encompassed lexical, syntactic, semantic, and pragmatic aspects of language. Commonly used linguistic features included lexical diversity measures (eg, type-token ratio), syntactic complexity metrics (eg, clausal density), semantic coherence measures (eg., idea density), and part-of-speech (POS) ratios.

Acoustic features focused on temporal characteristics of speech (eg, speech rate and pause patterns), spectral features (eg, mel-frequency cepstral coefficients), and voice quality measures (eg, jitter, shimmer). More recent studies have incorporated advanced NLP techniques such as word embeddings (eg, bidirectional encoder representations from transformers), n-gram language models, and deep learning architectures.

The included studies used various methods to compare linguistic and acoustic features between cognitively impaired and healthy groups, and to identify the most discriminative features or feature combinations. Common approaches included statistical tests (eg, *t* tests and ANOVAs) to compare individual features, machine learning classifiers (eg, support vector machines and random forests) to evaluate feature set performance, and feature selection techniques such as univariate methods, recursive feature elimination, and regularization. The optimal feature set was typically determined by evaluating model performance on held-out test data or through crossvalidation, with the combination of features yielding the highest accuracy or AUC considered the most discriminative.

Table 3^6^^,^^14^^,^^16^^,^^19^^,^^20^^,^^24^, ^25^, ^26^, ^27^, ^28^^,^^30^, ^31^, ^32^^,^^34^, ^35^, ^36^^,^^38^, ^39^, ^40^, ^41^^,^^44^^,^^46^, ^47^, ^48^, ^49^, ^50^, ^51^, ^52^^,^^57^^,^^61^^,^^62^ summarizes the importance of different feature types in cognitive impairment detection based on the included studies, along with their effect sizes, interpretation, advantages, and limitations. Linguistic features related to lexical diversity, syntactic complexity, and semantic coherence were found to be highly predictive across multiple studies. Acoustic features such as pause-related measures, speech rate, and spectral characteristics also recorded moderate to high importance. Combined approaches integrating both linguistic and acoustic features generally achieved the highest accuracy and AUC values.

**Table 3:** Importance of Different Feature Types in Cognitive Impairment Detection

Feature group	Specific measures	Studies	Effect size/importance	Interpretation
Speech timing and fluency	Pause-related measures (eg, pause duration, frequency)	^14^^,^^34^^,^^44^^,^^51^^,^^57^^,^^62^	High in multiple studies	Longer or more frequent pauses often indicate cognitive load or word-finding difficulties
	Speech rate	^14^^,^^34^^,^^44^^,^^51^	Moderate to high	Slower speech rate may indicate cognitive processing difficulties
	Repetitiveness	^35^^,^^48^	Moderate	Increased repetition may indicate memory or language impairment
	Verbal fluency measures	^50^	High in multiple studies	Reduced verbal fluency often associated with cognitive decline
Acoustic and prosodic features	Mel-frequency cepstral coefficients	^14^^,^^27^^,^^51^	Moderate to high	Captures spectral characteristics of speech, useful for detecting subtle changes in voice quality
	Fundamental frequency (F0)	^14^^,^^51^	Moderate	Changes in pitch patterns may indicate emotional or cognitive changes
	Acoustic-prosodic features (eg, rhythm and intonation)	^14^^,^^39^^,^^51^^,^^57^	Moderate to high	Changes in speech melody and rhythm can indicate cognitive changes
Lexical and semantic measures	Lexical diversity measures (eg, type-token ratio)	^14^^,^^16^^,^^25^^,^^28^^,^^34^^,^^51^^,^^61^	High in multiple studies	Lower diversity often associated with cognitive decline
	Word frequency/familiarity	^24^^,^^35^	Moderate	Use of more common words may increase with cognitive decline
	Semantic coherence/informativeness	^20^^,^^31^^,^^35^^,^^50^^,^^61^	High in multiple studies	Lower coherence or informativeness associated with cognitive decline
	Word embeddings (eg, BERT)	^19^^,^^27^^,^^32^^,^^41^^,^^49^	High in multiple studies	Captures semantic relationships, useful for detecting subtle language changes
	Content density	^16^^,^^48^	Moderate to high	Lower content density often associated with cognitive decline
Syntactic and structural complexity	Syntactic complexity	^16^^,^^38^^,^^47^^,^^48^	Moderate to high	Simpler syntactic structures may indicate cognitive decline
	Part-of-speech ratios	^14^^,^^38^^,^^47^^,^^52^	Moderate	Changes in distribution of word types can indicate language impairment
Conversational dynamics	Conversational features (eg, turn-taking)	^6^^,^^26^^,^^40^^,^^46^	Moderate to high	Changes in conversation dynamics can indicate cognitive or social changes
	Pronoun usage	^30^^,^^35^	Moderate	Changes in pronoun use may indicate difficulties with referencing
Technology-specific measures	n-Gram language models	^–^	Moderate to high	Captures local word dependencies, useful for detecting language patterns
	ASR error patterns	^41^	Moderate to high	ASR errors can be informative for detecting speech abnormalities
Feature selection and evaluation	Statistical tests (eg, *t* tests and ANOVAs), feature selection techniques (eg, univariate methods, recursive elimination, and regularization), model performance evaluation (eg, accuracy, AUC, and crossvalidation)	^14^^,^^16^^,^^24^^,^^25^^,^^27^^,^^28^^,^^34^, ^35^, ^36^^,^^38^^,^^47^, ^48^, ^49^, ^50^, ^51^, ^52^	High	Identifies most discriminative individual features and optimal feature combinations; evaluates model generalizability and robustness

ASR, automatic speech recognition; AUC, area under the curve; BERT, bidirectional encoder representations from transformers.

### Diagnostic Accuracy

It is important to note that most studies (∼80%) reported diagnostic accuracy by comparing NLP measures against established clinical diagnoses rather than reporting direct correlations with standardized cognitive test scores. Although some studies^14^^,^^20^^,^^31^ provided explicit correlations between their language measures and cognitive test scores (such as mini-mental state examination, Montreal cognitive assessment, or clinical dementia rating), most validated their approaches by showing how well NLP measures could match participants' existing clinical diagnostic groups. For instance, studies achieved high accuracy in distinguishing between clinically diagnosed groups: up to 99% accuracy for AD vs control,^19^ 94%-96% accuracy for MCI detection,^38^ and AUC values above 0.90 in several studies. Future research would benefit from reporting both diagnostic accuracy and specific correlations with standardized cognitive assessments to strengthen the validation of language-based cognitive screening tools.

Linguistic features, particularly those related to lexical diversity, syntactic complexity, and semantic coherence, were consistently found to be strong predictors of cognitive impairment across studies. Acoustic features, such as speech rate and pause patterns, also recorded high discriminative power. Studies using combined linguistic and acoustic approaches generally reported higher accuracy compared with single-modality analyses.

A detailed comparison of linguistic, acoustic, and combined analysis approaches revealed the strengths and limitations of each method. Linguistic feature analysis, used in 6 studies with an average sample size of 228, focused on measures like lexical diversity, syntactic complexity, semantic coherence, n-gram models, and POS patterns. These approaches had an average accuracy of 85.5% and an AUC of 0.86. Linguistic analysis is applicable to text data and less sensitive to recording quality, capturing high-level language processing. However, it misses prosodic and voice quality information and may overlook subtle speech changes.

Acoustic feature analysis, used in 2 studies with an average sample size of 143, examined measures such as speech rate, pauses, fundamental frequency, mel-frequency cepstral coefficients, jitter, and shimmer. These approaches had an average accuracy of 81.7% and an AUC of 0.83. Acoustic analysis detects subtle speech changes and is less influenced by education, showing potential for passive monitoring. However, it misses content information, is sensitive to recording conditions, and can be affected by noncognitive factors.

Combined approaches, integrating both linguistic and acoustic features, were used in 10 studies with an average sample size of 171. These methods achieved the highest performance, with an average accuracy of 87% and an AUC of 0.89. Combined analysis provides a comprehensive assessment, capturing both content and delivery aspects of speech. It is generally more accurate and robust across tasks. However, it requires both audio recordings and transcripts, involves a complex analysis pipeline, and is computationally intensive.

These findings suggest that integrating multiple modalities may provide the most comprehensive assessment of cognitive function. However, the increased complexity and computational demands of multimodal analyses should be weighed against their incremental benefits over single-modality approaches (Supplemental Table 2, available online at https://www.mcpdigitalhealth.org/).

### Crosslinguistic Analysis

Although all included studies were published in English, several studies analyzed speech data in other languages. The crosslinguistic analysis highlighted the applicability of NLP methods across different languages and cultural contexts. Studies analyzing speech in English served as a baseline for many investigations, with techniques adapted for other languages. The extensive NLP resources available in English and well-established markers facilitated the development of cognitive impairment detection models. However, potential Western education bias should be considered.

Studies in Chinese found high accuracy in MCI detection (94%-96%), emphasizing the importance of tonal features and language-specific syntax measures. Japanese studies reported high dementia detection accuracy (AUC, 0.935) and effective conversational analysis, considering factors like subject-object-verb word order, particle usage, and politeness levels.

Italian studies found acoustic features to be sensitive to cognitive decline and highlighted the role of rich morphology and cultural narrative styles. Turkish research achieved 83.5% accuracy using acoustic features, adapting to the agglutinative structure and vowel harmony of the language.

Thai studies using POS tagging and machine learning classification reported an AUC of 0.8480 for the best model, considering the tonal nature of the language and the use of classifiers in noun phrases. These findings underscore the potential for NLP techniques to be developed and validated in diverse linguistic settings, enabling global efforts in early detection of cognitive impairment (Supplemental Table 3, available online at https://www.mcpdigitalhealth.org/).

### Longitudinal vs Cross-sectional Designs

Of the 51 included studies, 5 used longitudinal designs, whereas 46 were cross-sectional. Longitudinal studies have the advantage of tracking individual changes over time and identifying markers predictive of future cognitive decline. They provide valuable insights into the progression of linguistic and acoustic features and have higher potential for early detection and monitoring. However, longitudinal studies are more resource-intensive, have smaller sample sizes (average, n=186), and may be affected by attrition and practice effects. Cross-sectional studies, on the contrary, allow for larger sample sizes (average n=312) and easier comparisons between diagnostic groups. They are useful for developing screening tools and identifying key differences between cognitively impaired and healthy individuals. However, cross-sectional designs cannot establish temporal relationships or track individual trajectories of decline.

The comparison of longitudinal and cross-sectional study designs revealed distinct strengths and limitations. Longitudinal studies had an average sample size of 159 participants and a study duration of 2-6 years, focusing on change over time. Key longitudinal studies detected subtle changes before clinical diagnosis, identified predictive markers of future decline, and reported acoustic and linguistic progression patterns.

Longitudinal designs have the advantage of tracking individual changes over time and identifying markers predictive of future cognitive decline. They provide valuable insights into the progression of linguistic and acoustic features and have higher potential for early detection and monitoring. Key predictive features in longitudinal studies included changes in syntactic complexity, decline in lexical diversity, increase in pause frequency/duration, and changes in acoustic features like fundamental frequency.

However, longitudinal studies are more resource-intensive, have smaller sample sizes, and may be affected by attrition and practice effects. They are promising for early intervention and personalized care plans but are time-consuming and costly to conduct.

Cross-sectional studies, on the contrary, had an average sample size of 274 participants and focused on group differences at a single time point. Key cross-sectional studies distinguished groups with high accuracy, identified key group differences, and provided snapshots at different impairment stages. They are useful for developing screening tools and identifying key differences between cognitively impaired and healthy individuals.

Cross-sectional designs allow for larger sample sizes and easier comparisons between diagnostic groups. Key predictive features in cross-sectional studies included vocabulary richness, syntactic complexity, semantic coherence, and acoustic features like speech rate and mel-frequency cepstral coefficients. These studies are easier to conduct and useful for diagnostic tools and treatment decisions.

However, cross-sectional designs cannot establish temporal relationships or track individual trajectories of decline. They may miss subtle changes and be affected by cohort effects, making them less predictive than longitudinal studies. Cross-sectional studies have a moderate potential for early detection, distinguishing early stages but potentially missing prodromal changes. They are not designed for monitoring cognitive decline over time (Supplemental Table 4, available online at https://www.mcpdigitalhealth.org/).

### Comparison of Linguistic, Acoustic, and Combined Approaches

Once a classifier is trained and validated, it can be used to predict the cognitive status of new individuals not part of the original data set. This process involves extracting the same set of features from the new individual’s speech sample, preprocessing the features using parameters derived from the training data, and applying the trained classifier to generate a predicted probability or class label. The prediction is then interpreted in the context of the model’s performance metrics and relevant clinical thresholds. However, the generalizability of a classifier depends on the diversity and representativeness of the training data, and models should be continuously monitored and updated to account for changes in language use, demographic characteristics, or diagnostic criteria over time.

The comparison of linguistic, acoustic, and combined analysis approaches revealed the strengths and limitations of each method. Linguistic feature analysis, used in 6 studies with an average sample size of 228, focused on measures like lexical diversity, syntactic complexity, semantic coherence, n-gram models, and POS patterns. These approaches had an average accuracy of 85.5% and an AUC of 0.86.

Linguistic analysis is applicable to text data and less sensitive to recording quality, capturing high-level language processing. However, it misses prosodic and voice quality information and may overlook subtle speech changes.

Acoustic feature analysis, used in 2 studies with an average sample size of 143, examined measures such as speech rate, pauses, fundamental frequency, mel-frequency cepstral coefficients, jitter, and shimmer. These approaches had an average accuracy of 81.7% and an AUC of 0.83.

Acoustic analysis detects subtle speech changes and is less influenced by education, showing potential for passive monitoring. However, it misses content information, is sensitive to recording conditions, and can be affected by noncognitive factors.

Combined approaches, integrating both linguistic and acoustic features, were used in 10 studies with an average sample size of 171. These methods achieved the highest performance, with an average accuracy of 87% and an AUC of 0.89.

Combined analysis provides a comprehensive assessment, capturing both content and delivery aspects of speech. It is generally more accurate and robust across tasks. However, it requires both audio recordings and transcripts, involves a complex analysis pipeline, and is computationally intensive (Supplemental Table 5, available online at https://www.mcpdigitalhealth.org/)

### Quality Assessment

The quality assessment of included studies using the customized tool is presented in Table 4.^4^, ^5^, ^6^^,^^14^^,^^16^^,^^17^^,^^19^^,^^20^^,^^22^, ^23^, ^24^, ^25^, ^26^, ^27^, ^28^, ^29^, ^30^, ^31^, ^32^, ^33^, ^34^, ^35^, ^36^, ^37^, ^38^, ^39^, ^40^, ^41^, ^42^, ^43^, ^44^, ^45^, ^46^, ^47^, ^48^, ^49^, ^50^, ^51^, ^52^, ^53^, ^54^, ^55^, ^56^, ^57^, ^58^, ^59^, ^60^, ^61^, ^62^, ^63^, ^64^ The quality assessment of included studies revealed that the overall study quality was rated as high for 10 studies, moderate to high for 27 studies, moderate for 10 studies, and low to moderate for 4 studies.

**Table 4:** Quality Assessment of Included Studies

Reference, year	Participant selection risk	Speech data risk	Analysis risk	Overall study quality rating
Huang et al,^14^ 2024	Low (community-based recruitment with clear inclusion criteria)	Moderate (potential background noise in recordings)	Low (appropriate statistical methods and machine learning techniques used)	Moderate to high
Diaz-Asper et al,^22^ 2022	Moderate (limited demographic diversity)	Low (standardized telephone recording procedure)	Low (comprehensive NLP and machine learning approach)	Moderate to high
Amini et al,^23^ 2023	Low (well-characterized Framingham Heart Study cohort)	Low (standardized NP test protocol)	Low (rigorous NLP and machine learning methods)	High
Beltrami et al,^16^ 2018	Moderate (clear inclusion criteria but potential education bias)	Low (standardized recording and transcription procedures)	Moderate (comprehensive feature analysis but lack of machine learning models)	Moderate
Fristed et al,^24^ 2022	Low (well-defined inclusion/exclusion criteria)	Low (standardized self-administered task)	Low (rigorous crossvalidation procedures)	High
Mahajan and Baths,^25^ 2021	Moderate (used standardized data set but limited demographic info)	Low (used standardized ADReSS data set)	Low (comprehensive model comparisons and evaluations)	Moderate to high
Nasreen et al,^26^ 2021	Moderate (matched age ranges, but limited to moderate-stage AD)	Low (naturalistic conversations, but from existing data set)	Low (rigorous statistical analysis and machine learning approaches)	Moderate to high
Haulcy and Glass,^27^ 2021	Low (age-matched and gender-matched groups)	Low (standardized elicitation task)	Moderate (complex ML pipeline, some risk of overfitting)	Moderate to high
Igarashi and Nihei,^28^ 2022	Moderate (small sample size, especially for MCI group)	Low (multiple tasks, standardized procedures)	Low (use of established ML methods, crossvalidation)	Moderate
Kleiman and Galvin,^29^ 2024	Moderate (unequal group sizes, age differences)	Low (multiple standardized tasks)	Low (comprehensive approach)	Moderate to high
Robin et al,^30^ 2023	Low (well-defined inclusion criteria)	Low (standardized interviews)	Low (appropriate statistical methods)	Moderate to high
Horigome et al,^31^ 2022	Low (clear inclusion/exclusion criteria)	Low (standardized 10-min conversation)	Low (rigorous machine learning approach with crossvalidation)	High
Liu et al,^32^ 2021	Unclear (used existing data set)	Low (used established picture description task)	Low (used crossvalidation and compared with other methods)	Unable to determine comprehensively due to limited reporting
Ntracha et al,^33^ 2020	Moderate (specific inclusion criteria)	Low (natural typing on own devices)	Low (multiple models and validation approaches used)	Moderate
Badal et al,^34^ 2024	Moderate (limited generalizability)	Low (standardized interview procedure, but full details not provided)	Low to moderate (comprehensive analysis, but lacks independent validation)	Moderate
Hernández-Domínguez et al,^35^ 2018	Low (well-defined groups)	Low (standardized task)	Low (comprehensive approach)	High
Orimaye et al,^36^ 2018	Moderate (limited demographic information provided)	Low (standardized picture description task used)	Low (advanced NLP and machine learning techniques used)	Moderate
Parsapoor et al,^37^ 2023	High (small, convenience sample)	Moderate (standardized tasks but limited details on recording quality)	Low (comprehensive feature analysis and machine learning approach)	Moderate
Wang et al,^38^ 2021	Low to moderate (education level differences)	Low (standardized recording procedures)	Low (rigorous NLP and ML methods)	Moderate to high
Runde et al,^19^ 2024	Moderate (limited demographic information provided)	Low (used established Pitt Corpus database)	Low to moderate (comprehensive analysis, but some limitations in data augmentation)	Moderate to high
Lindsay et al,^39^ 2021	Moderate (potential education bias)	Low (standardized recording and transcription procedures)	Low (rigorous cross-language validation)	Moderate to high
Mirheidari et al,^5^ 2024	Low (consecutive stroke survivors recruited)	Low (standardized data collection procedure)	Low (appropriate machine learning techniques used)	Good
de Arriba-Pérez et al,^40^ 2023	Moderate (sample from a specific region and association)	Low (automated collection through chatbot system)	Low (use of established NLP and machine learning techniques)	Moderate to good
Li et al,^41^ 2024	Low (used established data sets)	Moderate (poor audio quality in ADReSS data set)	Low (used established models and evaluation methods)	Moderate to high
Skirrow et al,^42^ 2022	Moderate (potential selection bias due to technology requirements)	Low (standardized automated collection procedure)	Low (automated analysis with established NLP techniques)	Moderate to high
de Arriba-Pérez et al,^43^ 2024	Unclear (limited information on participant selection)	Low (used natural dialog interactions)	Low (used multiple machine learning models and feature selection techniques)	Moderate
Roark et al,^44^ 2011	Low (well-defined groups based on CDR scores)	Low (standardized elicitation method)	Moderate (complex analysis methods with potential for overfitting)	Moderate to high
Amini et al,^45^ 2024	Low (well-established Framingham Heart Study cohort)	Low (standardized neuropsychological test interviews)	Low (rigorous machine learning methodology with crossvalidation)	High
de Arriba-Pérez and García-Méndez,^46^ 2024	Unclear (limited demographic information and potential biases of language models)	Low (standardized collection)	Low (robust ML methodology)	Moderate to high
Šubert et al,^47^ 2023	Low (matched controls)	Moderate (short recordings, potential transcription errors)	Low (automated analysis with manual validation)	Moderate to high
Fraser et al,^48^ 2016	Moderate (groups not matched for age and education)	Low (standardized picture description task)	Low (comprehensive linguistic analysis and machine learning approach)	Moderate to high
Kim et al,^49^ 2024	Moderate (imbalanced group sizes)	Low (standardized elicitation method)	Low (robust ML techniques and validation procedures)	Moderate to high
Gómez-Valadés et al,^50^ 2024	Low (well-defined inclusion criteria)	Low (standardized semantic fluency tasks)	Low (comprehensive ML approach with multiple models)	High
Hajjar et al,^51^ 2023	Moderate (potential misclassification issues)	Low to moderate	Moderate (potential overfitting)	Moderate
Sangchocanonta et al,^52^ 2021	Moderate (age differences between groups)	Low (culturally relevant tasks, validated transcription)	Low (multiple ML models, crossvalidation)	Moderate to good
Yan et al,^53^ 2024	Moderate (limited diversity in sample)	Low (natural interactions with SVA)	Low to moderate (novel MR-NLP approach with human validation)	Moderate
Kalpana Chowdary et al,^54^ 2025	High (very limited sample)	Moderate (automated collection, but potential for errors)	High (limited analysis details)	Low to moderate
Liang et al,^55^ 2022	Moderate (limited demographic information and representation)	Low (standardized data collection procedure)	Low (appropriate ML techniques and validation used)	Moderate
Soroski et al,^56^ 2022	Moderate (convenience sampling from memory clinic and community)	Low (standardized tasks, recorded audio)	Low (appropriate NLP and ML techniques used)	Moderate
Khodabakhsh et al,^57^ 2015	Low (age-matched, education-matched, and gender-matched controls)	Low (standardized recording procedure)	Moderate (multiple statistical tests without correction for comparisons)	Moderate to high
Sigona et al,^58^ 2025	Moderate (convenience sample from nursing homes)	Moderate (naturalistic conversations, but manual transcription)	Low (multiple analysis techniques applied)	Moderate to high
Anmella et al,^6^ 2024	Low (clear inclusion/exclusion criteria)	Low (standardized recording)	Unclear (full analysis not yet conducted)	NA (protocol)
Yeung et al,^20^ 2021	Low (well-defined inclusion criteria)	Low (standardized picture description task)	Moderate (small sample size limits statistical power)	Moderate
Reeves et al,^59^ 2020	Moderate (convenience sample from existing cohort)	Low (standardized video description task)	Low (established NLP method used)	Moderate
Stille et al,^60^ 2019	NA (computational model)	NA (simulated data)	Moderate (novel modeling approach, limited validation against human data)	Moderate
Kong et al,^61^ 2023	Moderate (community-based sample, but potential selection bias)	Moderate (short samples)	Low (established linguistic analysis methods used)	Moderate
Pistono et al,^62^ 2016	Low (clear inclusion/exclusion criteria)	Low (standardized elicitation task)	Low (appropriate statistical analyses)	Good
Ferrario et al,^63^ 2022	Low (community-dwelling older adults recruited through multiple channels)	Low (naturalistic observation using validated EAR method)	Moderate (appropriate machine learning techniques used, but limited sample size)	Moderate
Panesar and Pérez Cabello de Alba,^17^ 2023	High (very small, convenience sample)	Moderate (used existing validated data set but small sample)	High (manual analysis on small sample)	Low
Williams et al,^64^ 2021	Moderate (small, homogeneous sample)	Low (standardized collection methods)	Moderate (appropriate statistical methods, but limited by sample size)	Moderate
Aramaki et al,^4^ 2019	Moderate (potential selection bias in recruitment)	Moderate (variability in speech topics, personality influences)	Moderate (limited to basic statistical analyses, no advanced ML techniques)	Moderate

AD, Alzheimer disease; CDR, clinical dementia rating; MCI, mild cognitive impairment; ML, machine learning; MR-NLP, modified rule–based natural language processing; NLP, natural language processing; NA, not applicable; NP, neuropsychological protocol; ADReSS, Alzheimer's Dementia Recognition through Spontaneous Speech; SVA, smart voice assistant.

In terms of participant selection, 16 studies were rated as having low risk of bias, 28 as moderate, and 7 as high. Speech data collection methods were rated as low risk in 37 studies, moderate in 12, and unclear in 2. Analysis methods were rated as low risk in 40 studies, moderate in 9, high in 1, and unclear in 1.

Common sources of potential bias included small or unbalanced sample sizes, lack of detailed demographic information, and limited diversity in participant characteristics. Some studies also had potential confounding factors or used data collection methods that may have introduced variability or noise.

## Discussion

This systematic review synthesized the current state of research on NLP techniques for early detection of cognitive impairment from speech and language data. The included studies report the potential of linguistic and acoustic features in distinguishing between cognitively impaired and healthy individuals with high accuracy across a wide range of health conditions.

The crosslinguistic analysis highlights the applicability of NLP methods across different languages and cultural contexts. Although language-specific adaptations are necessary to capture unique features of cognitive impairment, the core principles of NLP-based assessment appear to be generalizable. This finding underscores the potential for NLP techniques to be developed and validated in diverse linguistic settings, enabling global efforts in early detection of cognitive impairment.

Longitudinal studies, although limited in number, provide valuable insights into the progression of linguistic and acoustic markers over time. They have the potential to identify early signs of cognitive decline and inform personalized monitoring and intervention strategies. Future research should prioritize larger, multisite longitudinal studies to establish the predictive validity and clinical utility of NLP-based approaches.

The comparative analysis of linguistic, acoustic, and combined approaches suggests that integrating multiple modalities may yield the highest diagnostic performance. Combined approaches can capture both content and delivery aspects of speech, providing a more comprehensive assessment of cognitive function. However, the increased complexity and computational demands of multimodal analyses should be weighed against their incremental benefits over single-modality approaches.

The clinical applicability of NLP techniques for early detection of cognitive impairment is promising but requires further investigation. Integration into routine health care settings would necessitate the development of standardized, user-friendly tools that can be easily administered and interpreted by health care providers. Ethical considerations, such as data privacy and informed consent, must also be addressed when deploying NLP-based assessments in clinical practice.

The potential role of NLP techniques in differential diagnosis of various types of cognitive impairment emerged as an important consideration from our review. Although most studies focused on distinguishing between healthy controls and individuals with cognitive impairment, several studies suggested that linguistic and acoustic markers might help differentiate between distinct pathologic conditions. For example, the analysis of semantic features could be particularly relevant in detecting AD, which typically presents with greater semantic impairment, whereas temporal acoustic measures might be more sensitive to the psychomotor slowing characteristic of PD dementia. Studies analyzing pause patterns, speech rate, and voice quality measures found high sensitivity to motor aspects of speech production, potentially offering distinctive markers for conditions like PD. However, research specifically comparing linguistic profiles across different types of dementia remains limited. Future studies should explore how specific combinations of NLP features might support differential diagnosis, particularly in distinguishing between conditions with overlapping clinical presentations but distinct underlying pathologies. This could be especially valuable in early stages when traditional clinical differentiation is challenging.

Several limitations of the current evidence base should be acknowledged. The heterogeneity of study designs, participant characteristics, speech elicitation methods, and NLP techniques hinders direct comparisons and meta-analyses. Many studies had small sample sizes and lacked diversity in terms of race, ethnicity, and education level. Most studies were conducted in controlled research settings, and the generalizability of findings to real-world clinical contexts remains to be established.

Future research should focus on standardizing speech elicitation protocols, harmonizing feature sets, and validating NLP-based approaches in larger, more diverse populations. The development of open-source, language-agnostic NLP toolkits could facilitate crosslinguistic validation and collaboration. Longitudinal studies with longer follow-up periods are needed to establish the predictive value of NLP markers and their ability to detect cognitive impairment at the earliest stages.

The quality assessment of included studies revealed some common sources of potential bias, such as small or unbalanced sample sizes, lack of detailed demographic information, and limited diversity in participant characteristics. Future studies should aim to minimize these biases by recruiting larger, more representative samples and providing clear descriptions of participant characteristics and data collection methods.

## Conclusion

This systematic review highlighted the potential of NLP techniques for early detection of cognitive impairment across a wide range of health conditions. Although promising, the field would benefit from larger, more diverse, and longitudinal studies to establish the robustness, generalizability, and clinical utility of NLP-based approaches. With continued research and development, NLP methods may provide a valuable tool for timely diagnosis, monitoring, and intervention in cognitive impairment, ultimately improving outcomes for affected individuals and their families.

## Potential Competing Interests

The authors report no competing interests.

## Supplemental Online Material

Supplementary Material

PRISMA_2020_checklist

## References

[1] Eshkoor S.A., Hamid T.A., Mun C.Y., Ng C.K. (2015). Mild cognitive impairment and its management in older people. Clin Interv Aging.

[2] Kasper S., Bancher C., Eckert A. (2020). Management of mild cognitive impairment (MCI): the need for national and international guidelines. World J Biol Psychiatry.

[3] Parra M.A., Butler S., McGeown W.J., Brown Nicholls L.A., Robertson D.J. (2019). Globalising strategies to meet global challenges: the case of ageing and dementia. J Glob Health.

[4] Aramaki E., Miyabe M., Honda C. (2019). KOTOBAKARI study: using natural language processing of patient short narratives to detect cancer related cognitive impairment. Stud Health Technol Inform.

[5] Mirheidari B., Bell S.M., Harkness K., Blackburn D., Christensen H. (2024). Spoken language-based automatic cognitive assessment of stroke survivors. Lang Health.

[6] Anmella G., De Prisco M., Joyce J.B. (2024). Automated speech analysis in bipolar disorder: the CALIBER study protocol and preliminary results. J Clin Med.

[7] Dubois B., Hampel H., Feldman H.H. (2016). Preclinical Alzheimer’s disease: definition, natural history, and diagnostic criteria. Alzheimers Dement.

[8] Zhao Q., Du X., Chen W., Zhang T., Xu Z. (2023). Advances in diagnosing mild cognitive impairment and Alzheimer’s disease using 11C-PIB- PET/CT and common neuropsychological tests. Front Neurosci.

[9] Sullivan V., Majumdar B., Richman A., Vinjamuri S. (2012). To scan or not to scan: neuroimaging in mild cognitive impairment and dementia. Adv Psychiatr Treat.

[10] Alzola P., Carnero C., Bermejo-Pareja F. (2024). Neuropsychological assessment for early detection and diagnosis of dementia: current knowledge and new insights. J Clin Med.

[11] Lee Y.-S., Youn H., Jeong H.-G. (2021). Cost-effectiveness of using amyloid positron emission tomography in individuals with mild cognitive impairment. Cost Eff Resour Alloc.

[12] Whelan R., Barbey F.M., Cominetti M.R., Gillan C.M., Rosická A.M. (2022). Developments in scalable strategies for detecting early markers of cognitive decline. Transl Psychiatry.

[13] Pourramezan Fard A., Mahoor M., Alsuhaibani M., Dodge H. (2024). Linguistic-based mild cognitive impairment detection using informative loss. Comput Biol Med.

[14] Huang L., Yang H., Che Y., Yang J. (2024). Automatic speech analysis for detecting cognitive decline of older adults. Front Public Health.

[15] Lefkovitz I., Walsh S., Blank L.J., Jetté N., Kummer B.R. (2024). Direct clinical applications of natural language processing in common neurological disorders: scoping review. JMIR Neurotech.

[16] Beltrami D., Gagliardi G., Rossini Favretti R. (2018). Speech analysis by natural language processing techniques: a possible tool for very early detection of cognitive decline?. Front Aging Neurosci.

[17] Panesar K., Pérez Cabello de Alba M.B. (2023). Natural language processing-driven framework for the early detection of language and cognitive decline. Lang Health.

[18] Jahan Z., Khan S.B., Saraee M. (2024). Early dementia detection with speech analysis and machine learning techniques. Discov Sustain.

[19] Runde B.S., Alapati A., Bazan N.G. (2024). The optimization of a natural language processing approach for the automatic detection of Alzheimer’s disease using GPT embeddings. Brain Sci.

[20] Yeung A., Iaboni A., Rochon E. (2021). Correlating natural language processing and automated speech analysis with clinician assessment to quantify speech-language changes in mild cognitive impairment and Alzheimer’s dementia. Alzheimers Res Ther.

[21] Page M.J., McKenzie J.E., Bossuyt P.M. (2021). The PRISMA 2020 statement: an updated guideline for reporting systematic reviews. BMJ.

[22] Diaz-Asper C., Chandler C., Turner R.S., Reynolds B., Elvevåg B. (2022). Increasing access to cognitive screening in the elderly: applying natural language processing methods to speech collected over the telephone. Cortex.

[23] Amini S., Hao B., Zhang L. (2023). Automated detection of mild cognitive impairment and dementia from voice recordings: a natural language processing approach. Alzheimers Dement.

[24] Fristed E., Skirrow C., Meszaros M. (2022). A remote speech-based AI system to screen for early Alzheimer's disease via smartphones. Alzheimers Dement (Amst).

[25] Mahajan P., Baths V. (2021). Acoustic and language based deep learning approaches for Alzheimer’s dementia detection from spontaneous speech. Front Aging Neurosci.

[26] Nasreen S., Rohanian M., Hough J., Purver M. (2021). Alzheimer’s dementia recognition from spontaneous speech using disfluency and interactional features. Front Comput Sci.

[27] Haulcy R.M., Glass J. (2021). Classifying Alzheimer’s disease using audio and text-based representations of speech. Front Psychol.

[28] Igarashi T., Nihei M. (2022). Cognitive assessment of Japanese older adults with text data augmentation. Healthcare (Basel).

[29] Kleiman M.J., Galvin J.E. High frequency post-pause word choices and task-dependent speech behavior characterize connected speech in individuals with mild cognitive impairment. Preprint. http://10.1101/2024.02.25.24303329.

[30] Robin J., Xu M., Balagopalan A. (2023). Automated detection of progressive speech changes in early Alzheimer’s disease. Alzheimers Dement (Amst).

[31] Horigome T., Hino K., Toyoshiba H. (2022). Identifying neurocognitive disorder using vector representation of free conversation. Sci Rep.

[32] Liu N., Yuan Z., Tang Q. (2021). Improving Alzheimer’s disease detection for speech based on feature purification network. Front Public Health.

[33] Ntracha A., Iakovakis D., Hadjidimitriou S. (2020). Detection of mild cognitive impairment through natural language and touchscreen typing processing. Front Digit Health.

[34] Badal V.D., Reinen J.M., Twamley E.W. (2024). Investigating acoustic and psycholinguistic predictors of cognitive impairment in older adults: modeling study. JMIR Aging.

[35] Hernández-Domínguez L., Ratté S., Sierra-Martínez G., Roche-Bergua A. (2018). Computer-based evaluation of Alzheimer’s disease and mild cognitive impairment patients during a picture description task. Alzheimers Dement (Amst).

[36] Orimaye S.O., Wong J.S., Wong C.P. (2018). Deep language space neural network for classifying mild cognitive impairment and Alzheimer-type dementia. PLoS One.

[37] Parsapoor Parsa MM., Alam M.R., Mihailidis A. (2023). Performance of machine learning algorithms for dementia assessment: impacts of language tasks, recording media, and modalities. BMC Med Inform Decis Mak.

[38] Wang T., Hong Y., Wang Q. (2021). Identification of mild cognitive impairment among Chinese based on multiple spoken tasks. J Alzheimers Dis.

[39] Lindsay H., Tröger J., König A. (2021). Language impairment in Alzheimer’s disease-robust and explainable evidence for AD-related deterioration of spontaneous speech through multilingual machine learning. Front Aging Neurosci.

[40] de Arriba-Pérez F., García-Méndez S., González-Castaño F.J., Costa-Montenegro E. (2023). Automatic detection of cognitive impairment in elderly people using an entertainment chatbot with natural language processing capabilities. J Ambient Intell Humaniz Comput.

[41] Li C., Xu W., Cohen T., Pakhomov S. (2024). Useful blunders: can automated speech recognition errors improve downstream dementia classification?. J Biomed Inform.

[42] Skirrow C., Meszaros M., Meepegama U. (2022). Validation of a remote and fully automated story recall task to assess for early cognitive impairment in older adults: longitudinal case-control observational study. JMIR Aging.

[43] de Arriba-Pérez F., García-Méndez S., Otero-Mosquera J., González-Castaño F.J. (2024). Explainable cognitive decline detection in free dialogues with a machine learning approach based on pre-trained large language models. Appl Intell.

[44] Roark B., Mitchell M., Hosom J.P., Hollingshead K., Kaye J. (2011). Spoken language derived measures for detecting mild cognitive impairment. IEEE Trans Audio Speech Lang Process.

[45] Amini S., Hao B., Yang J. (2024). Prediction of Alzheimer’s disease progression within 6 years using speech: a novel approach leveraging language models. Alzheimers Dement.

[46] de Arriba-Pérez F., García-Méndez S. (2024). Leveraging large language models through natural language processing to provide interpretable machine learning predictions of mental deterioration in real time. Arab J Sci Eng.

[47] Šubert M., Novotný M., Tykalová T. (2023). Lexical and syntactic deficits analyzed via automated natural language processing: the new monitoring tool in multiple sclerosis. Ther Adv Neurol Disord.

[48] Fraser K.C., Meltzer J.A., Rudzicz F. (2016). Linguistic features identify Alzheimer’s disease in narrative speech. J Alzheimers Dis.

[49] Kim H., Hillis A.E., Themistocleous C. (2024). Machine learning classification of patients with amnestic mild cognitive impairment and non-amnestic mild cognitive impairment from written picture description tasks. Brain Sci.

[50] Gómez-Valadés A., Martínez R., Rincón M. (2024). Designing an effective semantic fluency test for early MCI diagnosis with machine learning. Comput Biol Med.

[51] Hajjar I., Okafor M., Choi J.D. (2023). Development of digital voice biomarkers and associations with cognition, cerebrospinal biomarkers, and neural representation in early Alzheimer’s disease. Alzheimers Dement (Amst).

[52] Sangchocanonta S., Vongsurakrai S., Sroykhumpa K. (2021). Development of Thai picture description task for Alzheimer’s screening using part-of-speech tagging. Annu Int Conf IEEE Eng Med Biol Soc.

[53] Yan Z., Dube V., Heselton J. (2024). Understanding older people’s voice interactions with smart voice assistants: a new modified rule-based natural language processing model with human input. Front Digit Health.

[54] Kalpana Chowdary M., Gopatoti A., Ferlin Deva Shahila D. (2025). Entertainment robots for automatic detection and mitigation of cognitive impairment in elderly populations. Entertain Comput.

[55] Liang X., Batsis J.A., Zhu Y. (2022). Evaluating voice-assistant commands for dementia detection. Comput Speech Lang.

[56] Soroski T., da Cunha Vasco T., Newton-Mason S. (2022). Evaluating web-based automatic transcription for Alzheimer speech data: transcript comparison and machine learning analysis. JMIR Aging.

[57] Khodabakhsh A., Yesil F., Guner E., Demiroglu C. (2015). Evaluation of linguistic and prosodic features for detection of Alzheimer’s disease in Turkish conversational speech. EURASIP J Audio Speech Music Process.

[58] Sigona F., Radicioni D.P., Gili Fivela B. (2025). A computational analysis of transcribed speech of people living with dementia: the Anchise 2022 Corpus. Comput Speech Lang.

[59] Reeves S., Williams V., Costela F.M. (2020). Narrative video scene description task discriminates between levels of cognitive impairment in Alzheimer’s disease. Neuropsychology.

[60] Stille C.M., Bekolay T., Blouw P., Kröger B.J. (2019). Natural language processing in large-scale neural models for medical screenings. Front Robot AI.

[61] Kong A.P.-H., Cheung R.T.H., Wong G.H.Y. (2023). Spoken discourse in episodic autobiographical and verbal short-term memory in Chinese people with dementia: the roles of global coherence and informativeness. Front Psychol.

[62] Pistono A., Jucla M., Barbeau E.J. (2016). Pauses during autobiographical discourse reflect episodic memory processes in early Alzheimer’s disease. J Alzheimers Dis.

[63] Ferrario A., Luo M., Polsinelli A.J. (2022). Predicting working memory in healthy older adults using real-life language and social context information: a machine learning approach. JMIR Aging.

[64] Williams K., Myers J.S., Hu J., Manson A., Maliski S.L. (2021). Psycholinguistic screening for cognitive decline in cancer survivors: a feasibility study. Oncol Nurs Forum.

