# Supplementary material for: A Systematic Review of Natural Language Processing Techniques for Early Detection of Cognitive Impairment
Source: Mayo Clin Proc Digit Health. 2025 Mar 5;3(2):100205. doi: 10.1016/j.mcpdig.2025.100205 (PMC12190899; doi:10.1016/j.mcpdig.2025.100205)
Supplement: Supplementary Material [file mmc1.docx]

**Supplementary Material**

Table S1: Data Extraction Variables

| **Category** | **Variables** |
| --- | --- |
| Study Characteristics | - First author  - Publication year  - Country  - Language of speech data  - Study design |
| Participant Characteristics | - Sample size  - Participant groups  - Age (mean, range)  - Gender  - Education level  - Comorbidities |
| Speech Elicitation Methods | - Task description  - Standardization  - Duration  - Recording equipment |
| NLP Techniques | - Linguistic features  - Acoustic features  - Machine learning algorithms  - Feature selection methods |
| Outcomes | - Diagnostic accuracy metrics (e.g., accuracy, AUC, sensitivity, specificity)  - Key predictive features  - Comparison of approaches |
| Limitations | - Sample size and representativeness  - Confounding factors  - Generalizability  - Technical limitations |

Table S2: Speech and Language Markers Across Different Cognitive Disorders

| **Feature Category** | **Feature** | **MCI** | **AD** | **Other Neurological Disorders** |
| --- | --- | --- | --- | --- |
| Lexical | Vocabulary richness (e.g., type-token ratio) | ✓✓✓ | ✓✓✓ | ✓✓ (MS, PD) |
|  | Word frequency/familiarity | ✓✓ | ✓✓✓ | ✓ (PD) |
|  | Noun/verb ratio | ✓✓ | ✓✓✓ | ✓ (PD) |
|  | Use of function words | ✓ | ✓✓ | ✓ (MS) |
| Syntactic | Syntactic complexity | ✓✓✓ | ✓✓ | ✓✓ (MS, PD) |
|  | Dependency distance | ✓✓ | ✓✓ | ✓ (MS) |
|  | Phrase length | ✓✓ | ✓✓✓ | ✓ (PD) |
| Semantic | Idea density | ✓✓✓ | ✓✓✓ | ✓ (PD) |
|  | Semantic coherence | ✓✓ | ✓✓✓ | ✓✓ (MS, PD) |
|  | Information content | ✓✓ | ✓✓✓ | ✓ (PD) |
| Pragmatic | Topic maintenance | ✓✓ | ✓✓✓ | ✓ (PD) |
|  | Repetitiveness | ✓ | ✓✓✓ | ✓✓ (PD, MS) |
|  | Turn-taking behavior | ✓ | ✓✓ | ✓✓ (PD) |
| Acoustic (Temporal) | Speech rate | ✓✓✓ | ✓✓✓ | ✓✓✓ (PD, MS, Stroke) |
|  | Pause frequency and duration | ✓✓✓ | ✓✓✓ | ✓✓✓ (PD, MS, Stroke) |
|  | Hesitations | ✓✓ | ✓✓✓ | ✓✓ (PD, Stroke) |
| Acoustic (Spectral) | Fundamental frequency (F0) | ✓✓ | ✓✓ | ✓✓✓ (PD, MS) |
|  | Mel-frequency cepstral coefficients (MFCCs) | ✓✓✓ | ✓✓✓ | ✓✓✓ (PD, MS, Stroke) |
|  | Formant frequencies | ✓✓ | ✓✓ | ✓✓✓ (PD, MS) |
| Acoustic (Voice Quality) | Jitter | ✓ | ✓✓ | ✓✓✓ (PD) |
|  | Shimmer | ✓ | ✓✓ | ✓✓✓ (PD) |
|  | Harmonics-to-noise ratio | ✓ | ✓✓ | ✓✓✓ (PD) |

*Legend: ✓✓✓ Highly predictive across multiple studies, ✓✓ Moderately predictive or predictive in some studies, ✓ Some evidence of predictive value; MCI: Mild Cognitive Impairment, AD: Alzheimer's Disease, MS: Multiple Sclerosis, PD: Parkinson's Disease*

Table S3: Cross-Linguistic Analysis of Speech-Based Cognitive Impairment Detection

| **Language** | **Studies** | **Sample Size** | **Key NLP Techniques** | **Main Findings** | **Language/Culture-Specific Considerations** | **Generalizability Insights** |
| --- | --- | --- | --- | --- | --- | --- |
| English | ^16,19,24-27,34,36,39,44^ | Range: 74-517, Avg: 246 | N-gram models, syntactic complexity, word embeddings, acoustic analysis | High accuracy in AD/MCI detection (AUC 0.80-0.95); Syntactic complexity and lexical diversity highly predictive | Extensive NLP resources; Well-established markers; Potential Western education bias | Baseline for many studies; Techniques adapted for other languages |
| Chinese | ^32,38^ | Range: 92-498, Avg: 295 | POS analysis, dependency parsing, acoustic features | High accuracy in MCI detection (94-96%); Importance of tonal features | Tonal language; Different syntax; Character-based analysis | Acoustic features important; Language-specific syntax measures |
| Japanese | ^4,28,31^ | Range: 60-432, Avg: 203 | Vector representations, type-token ratio | High dementia detection accuracy (AUC 0.935); Effective conversational analysis | SOV word order; Particle usage; Politeness levels | Conversational analysis effective; Language-specific grammar |
| Italian | ^16,58^ | Range: 96-216, Avg: 156 | Acoustic-prosodic features, information content | Acoustic features sensitive to cognitive decline; Verb usage patterns | Rich morphology; Cultural narrative styles | Acoustic features consistent; Morphological complexity |
| Turkish | ^57^ | 79 | Acoustic features, prosodic features | 83.5% accuracy with acoustic features | Agglutinative structure; Vowel harmony | Acoustic features applicable; Language-specific morphology |
| Thai | ^52^ | 90 | POS tagging, ML classification | AUC of 0.8480 for best model | Tonal language; Classifier usage in noun phrases | Tone analysis for diagnosis |

Table S4: Comparison of Longitudinal and Cross-sectional Studies Aspect

| **Aspect** | **Longitudinal Studies** | **Cross-sectional Studies** |
| --- | --- | --- |
| Number of Studies | 5 | 46 |
| Average Sample Size | 159 | 274 |
| Average Study Duration | 2-6 years | N/A |
| Primary Focus | Change over time | Group differences |
| Key Studies | ^30,42,45,63^ | ^16,23-25,32,38,39,48^ |
| Main Findings | Detected subtle changes before clinical diagnosis; Identified predictive markers of future decline; Showed acoustic and linguistic progression patterns | Distinguished groups with high accuracy; Identified key group differences; Provided snapshots at different impairment stages |
| Key Predictive Features | Changes in syntactic complexity; Decline in lexical diversity; Increasing pause frequency/duration; Changes in acoustic features (e.g., F0) | Vocabulary richness; Syntactic complexity; Semantic coherence; Acoustic features (e.g., speech rate, MFCCs) |
| Advantages | Can detect early, subtle changes; Allows prediction of future decline; Accounts for individual variability; Provides progression insights | Larger samples possible; Easier to conduct; Compares stages; Useful for diagnostic tools |
| Limitations | Time-consuming, resource-intensive; Higher dropout rates; Smaller samples; Potential practice effects | Cannot track individuals; May miss subtle changes; Cohort effects; Less predictive |
| Potential for Early Detection | High - Can detect preclinical changes | Moderate - Distinguishes early stages but may miss prodromal changes |
| Potential for Monitoring | High - Tracks changes over time | Low - Not designed for monitoring |
| Clinical Applicability | Promising for early intervention; Informs personalized care plans | Useful for screening and diagnosis; Informs treatment decisions; Stages cognitive impairment |

Table S5: Comparison of Linguistic, Acoustic, and Combined Analysis Approaches

| **Analysis Type** | **Studies** | **Average Sample Size** | **Average Accuracy/AUC** | **Key Features** | **Advantages** | **Limitations** |
| --- | --- | --- | --- | --- | --- | --- |
| Linguistic Features Only | ^25,28,36,48-50^ | 228 | Accuracy: 85.5% AUC: 0.86 | Lexical diversity, syntactic complexity, semantic coherence, n-gram models, POS patterns | Applicable to text data; Less sensitive to recording quality; Captures high-level language processing | Misses prosodic, voice quality info; May overlook subtle speech changes |
| Acoustic Features Only | ^51,57^ | 143 | Accuracy: 81.7% AUC: 0.83 | Speech rate, pauses, F0, MFCCs, jitter, shimmer | Detects subtle speech changes; Less influenced by education; Potential for passive monitoring | Misses content info; Sensitive to recording conditions; Affected by non-cognitive factors |
| Combined (Linguistic + Acoustic) | ^5,14,16,24,27,35,38,39,47^ | 171 | Accuracy: 87% AUC: 0.89 | Linguistic: Lexical diversity, syntactic complexity, semantic coherence; Acoustic: Speech rate, pauses, MFCCs, voice quality | Comprehensive analysis; Captures content and delivery; Generally higher accuracy; Robust across tasks | Requires audio and transcripts; Complex analysis pipeline; Computationally intensive |
